# Considerations on the TardiVec-based analyses of tissue specificity and desiccation-induced supramolecular structure of target proteins

**DOI:** 10.1073/pnas.2312563120

**Published:** 2023-11-20

**Authors:** Akihiro Tanaka, Takekazu Kunieda

**Affiliations:** ^a^Department of Biological Sciences, Graduate School of Science, The University of Tokyo, Tokyo 113-0033, Japan

Tardigrades are known for their remarkable ability to withstand extreme stresses (1). However, limited gene manipulation technology in tardigrades has hindered the elucidation of the tolerance mechanisms in vivo (2, 3). Recently, Tanaka et al. (4) developed a transient expression system for tardigrades using plasmid vectors named TardiVec (4). This technological advancement is undoubtedly important in tardigrade research, especially for live imaging. However, careful considerations are required when interpreting the visualized pattern of the expressed proteins.

**Fig. 1.**
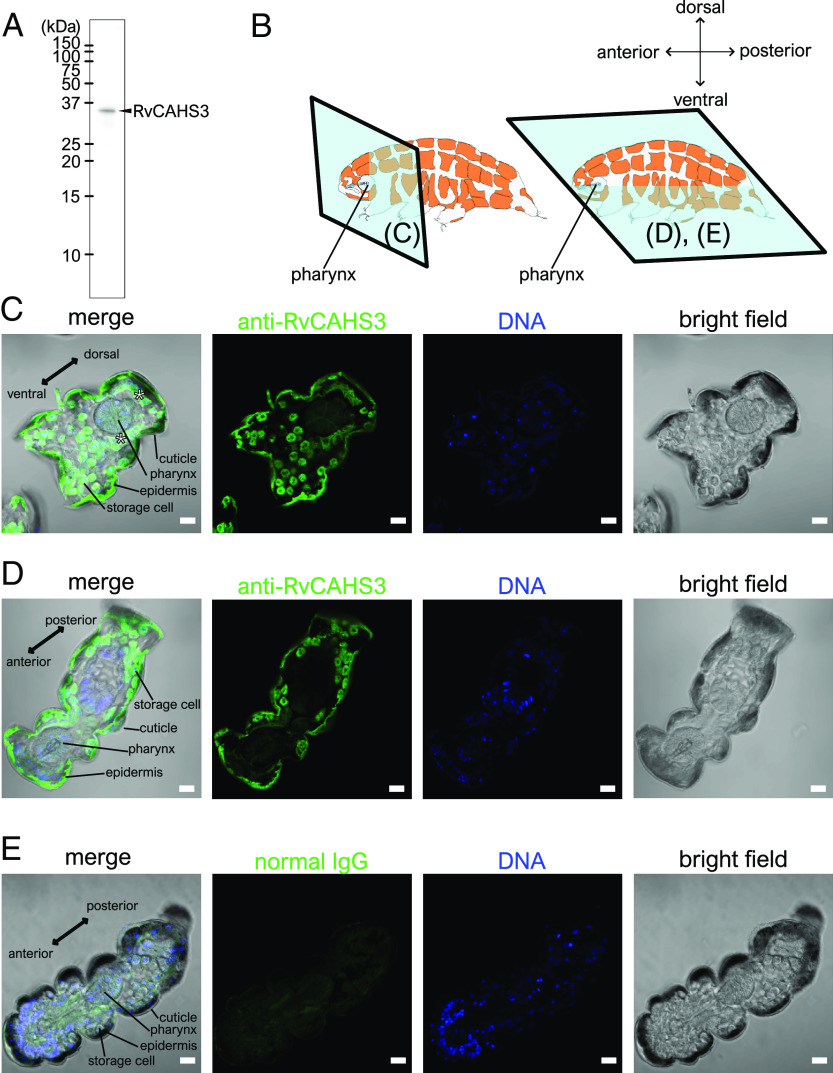
The endogenous RvCAHS3 protein is present in both epidermal and storage cells in *R. varieornatus*. (*A*) Specific detection of RvCAHS3 in *R. varieornatus* lysates using an anti-RvCAHS3 antibody in western blot analysis. Reproduced from the ref. 6. (*B*) An illustration of sectioning planes (blue squares) of tardigrade in each panel. (*C*–*E*) The representative images of immunohistochemistry on the thin sections of the tardigrades with the anti-RvCAHS3 antibody (*C* and *D*) or a normal IgG (*E*). Asterisks indicate unspecified tissues. The images were captured with a confocal microscope (Zeiss, LSM900). (Scale bar, 10 µm.)

TardiVec are vectors designed to express exogenous proteins, such as GFP, under the control of the ~1-kbp upstream sequence of the target genes. As demonstrated, this 1-kbp segment drives distinct tissue-selective expressions depending on the target genes (4). Correspondingly, the intrinsic tissue specificity of the target gene was discussed based on the results obtained using TardiVec, for instance, highlighting that one of the most studied desiccation-tolerance proteins, RvCAHS3 (4–6), was expressed only in epidermal cells. However, the endogenous expression pattern of the target proteins has not been confirmed. Here, we present immunohistochemical images of the RvCAHS3 on thin sections of adult *Ramazzottius varieornatus* using a previously established specific antibody (6) in Fig. 1*A*. The results revealed specific fluorescent signals not only in epidermal cells but also in storage cells (Fig. 1 *B*–*E*). This observation is consistent with the authors’ RNA-seq data indicating high expression (~1,000 tpm) of RvCAHS3 in storage cells (4). It is not surprising to find a discrepancy in tissue specificity between the TardiVec-based results and the endogenous protein because the expression of animal genes is generally regulated by both proximal cis-elements and distant elements known as enhancers (7). The TardiVec system could be used for screening the expression profiles of various genes, although additional examinations such as immunohistochemistry or tissue/cell-specific RNA-seq will be necessary to conclude their tissue specificity.

**Fig. 2.**
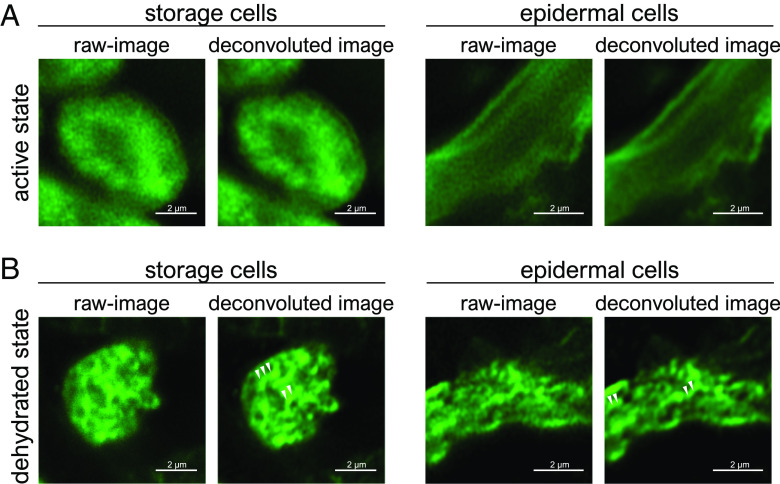
RvCAHS3 proteins form filaments in the desiccated tardigrade cells. The intracellular distribution of RvCAHS3 proteins in the hydrated or desiccated tardigrades was examined by immunocytochemistry using the anti-RvCAHS3 antibody. The images were captured using a confocal microscope with super-resolution processing (Zeiss, LSM980 with Airyscan2). (*A*) The broad distribution of RvCAHS3 protein in the cytosol of active hydrated tardigrade cells. (*B*) The filament structures of RvCAHS3 proteins were detected in both storage cells and epidermal cells of desiccated tardigrades. Deconvolution processing was performed to clarify the images. White arrows indicate the filamentous structures.

Using TardiVec provides another notable advantage to enable live imaging the in vivo distribution changes of target proteins in desiccating tardigrades. An earlier study has shown that RvCAHS3 form numerous filaments in both human and insect cells under water-deficient stress (6). Using TardiVec, however, the authors did not observe filament formation of CAHS3-GFP in a desiccating tardigrade, although they also noted the difficulty in observing the fluorescence in dehydrated tardigrades due to cell compression, insufficient light penetration, and possible change in refractive index. To address this, we captured fine immunocytochemical images on sections of desiccated tardigrades using high-resolution microscopy. The endogenous RvCAHS3 were broadly present in the cytosol of hydrated tardigrade cells but exhibited distinct filamentous structures in dehydrated tardigrade cells (Fig. 2). The differences between their results and ours may be attributed to the methodology of the observations, while we would not exclude other possibilities. They observed the fluorescence inside animals whose complexity could make the fluorescent signals blur resulting in a low resolution.

In conclusion, the expression system using TardiVec provides valuable opportunities for tardigrade research. It is also recommended to consider the limitations in recapitulating the endogenous expression pattern and the difficulty in observing fine structures in desiccated tardigrades. Confirmation using alternative methods will solidify the findings about these aspects.
